# The financial impact on people with coeliac disease of withdrawing gluten-free food from prescriptions in England: findings from a cross-sectional survey

**DOI:** 10.1186/s12913-024-10600-4

**Published:** 2024-01-30

**Authors:** Thavapriya Sugavanam, Helen Crocker, Mara Violato, Michele Peters

**Affiliations:** 1 Nuffield Department of Population Health, University of Oxford, Oxford, UK; 2 Oxford Centre for Diabetes, Endocrinology, and Metabolism (OCDEM), University of Oxford, Churchill Hospital, Headington, Oxford, OX3 7LE UK

**Keywords:** Gluten-free food, Coeliac disease, Prescription changes, Finance, Policy

## Abstract

**Background:**

A lifelong gluten-free diet is the only treatment for coeliac disease. The cost and availability of gluten-free substitute food (GFSF) remain challenging. Some local areas in England have stopped gluten-free prescriptions for coeliac disease. The aim of this paper is to present the quantitative findings of the financial impact of prescription withdrawal on people with coeliac disease.

**Methods:**

A cross-sectional survey with adults in England who reported having been diagnosed with coeliac disease by a health professional. The postal survey was distributed by Coeliac UK to their members in 13 prescribing and 13 non-prescribing local areas that were matched for geographical location and level of deprivation. Additionally, an advertisement for the survey was placed on social media. The questionnaire contained items on the availability and use of prescriptions; the weekly amount spent on GFSF; amount of specific GFSF bought; affordability of GFSF; demographics and health-related variables. Data were analysed by descriptive statistics, analysis of variance and regression analysis.

**Results:**

Of the 1697 participants, 809 resided in areas that provided prescriptions and 888 in non-prescribing areas. Participants self-report of their prescription did not always match the local area prescription policy. There was no statistically significant difference between prescribing and non-prescribing areas in how easy or difficult participants found it to obtain GFSF (*p* = 0.644) and its availability in various locations. Participants in non-prescribing areas purchased most types of GFSF items in statistically significantly higher quantities and thereby spent an additional £11.32/month on GFSF items than participants in prescribing areas (*p* < 0.001). While taking into account the self-reported prescription status, the amount increased to £14.09/month (*p* < 0.001). Although affordability to buy GFSF did not differ based on local area prescription policy or self-reported prescription status, it was dependent on equivalised annual income. However, affordability did not influence spending on GFSF. Regression analysis indicated that males and households with additional members with coeliac disease spent more on GFSF.

**Conclusions:**

The study has highlighted that gluten-free prescription withdrawal can have financial implications for people with coeliac disease. Any future changes to the prescription policy of GFSF should consider the impact on the population, especially lower income households.

**Supplementary Information:**

The online version contains supplementary material available at 10.1186/s12913-024-10600-4.

## Background

Coeliac disease is an autoimmune condition in which the immune response is caused by the ingestion of gluten, a major protein found in wheat, rye and barley [1]. Lifelong avoidance of these foods (i.e., a gluten-free diet) is currently the only treatment [1]. Better adherence to the diet leads to fewer symptoms [2] and improved quality of life [3–6], while non-adherence may lead to long-term complications such as osteoporosis and iron deficiency anaemia [7, 8]. However, following such a diet can be demanding and burdensome [9, 10]. Moreover, the cost, access and availability of gluten-free substitute food (GFSF) continues to be challenging [11–15]. Generally, costing considerably more than gluten-containing equivalent foods, GFSF bought online or in rural areas are even more expensive than those bought in supermarkets or urban areas [11, 14–19].

To cover the additional cost involved in following a gluten-free diet and to support people’s adherence to the diet, some countries, such as the United States, Canada and Italy [20], provide tax relief and government subsidisation. In England, GFSF was provided on prescription by the National Health Service (NHS), in accordance with National Prescription Guidelines, until 2015 [21]. Since 2015, due to the financial strain on the NHS, some local statutory bodies responsible for planning and commissioning services, called Clinical Commissioning Groups (CCGs), began to withdraw or restrict (lower quantity or fewer food types) prescriptions, creating variation in prescription policy throughout England [22, 23]. Although this change appeared to be cost-effective for the NHS [24], the restriction and withdrawal of prescriptions began to show negative impacts on dietary adherence [25]. There was also concern that regional differences may lead to inequality of access, given the variation in availability of GFSF [15].

The data presented in this paper are part of a mixed methods study to assess the impact of changes in prescription policy for GFSF for adults with coeliac disease living in England. The findings regarding the impact of prescription withdrawal on quality of life (quantitative and qualitative) and the impact on affordability and obtainability of GFSF (qualitative) have been published elsewhere [26, 27]. The aim of this paper is to present the quantitative findings of the financial impact of prescription withdrawal between people living in areas that stopped prescriptions and people living in areas that continued to provide prescriptions according to the National Prescription Guidelines [21]. Comparisons will be drawn for the following: (i) ease of access to GFSF, (ii) availability of GFSF, (iii) estimated spending on GFSF, (iv) self-reported spending on GFSF, (v) affordability to buy GFSF and (vi) equivalised income.

## Methods

A sequential explanatory mixed methods study was conducted including a cross-sectional survey, which was followed by qualitative interviews. The quantitative findings, from the cross-sectional survey, relating to of the financial impact of prescription withdrawal is presented in this paper. The Central University Research Ethics Committee of the University of Oxford approved the study (Reference number R45890/RE001). Signed informed consent was obtained from all participants at the beginning of the survey. All methods carried out in the study were performed in line with the approved protocol and relevant guidelines/regulations. The detailed methods have been reported elsewhere [26]. A summary of the survey methods is given below.

### Survey

Sample size calculations were undertaken using the sensitivity to change data for the Coeliac Disease Assessment Questionnaire (CDAQ), the questionnaire used to assess quality of life (see Peters et al. [26] for details). To achieve a sample of 800, a minimum of 2000 people had to be invited (40% estimated response rate). To allow for the inclusion of a wider geographical spread of local areas, a larger than necessary sample was recruited.

A total of 4050 members were invited into a postal survey by Coeliac UK, the leading charity for coeliac disease in the UK. Members were selected from 13 local areas (or CCGs) that prescribed GFSF according to the National Prescribing Guidance (*n* = 2131) and from 13 local areas that had stopped prescriptions (*n* = 1919). Matched for geographical location and level of deprivation, local areas were selected to achieve a spread of urban and rural communities across England. Coeliac UK selected members from their database in the relevant geographical regions. The aim was to invite 200 members per local area. However, membership varied by area (range 10–624 members), and therefore, in areas (*n* = 18) with fewer than 200 members, all members were invited. In the eight areas with more than 200 members, Coeliac UK randomly selected members for the study. As the survey was distributed to Coeliac UK members, the representativeness of the participants to the region (e.g., ethnicity, level of deprivation) could not be taken into consideration.

In addition to the postal survey, an online advertisement for the survey was placed on social media sites Twitter and Facebook. The advertisement included a link to an electronic version of the survey (e-survey) using Qualtrics software, Version April 2017 (Qualtrics, Provo, UT).

The inclusion criteria to participate in the survey were: (i) adults (18 years of age or above), (ii) live in England, and (iii) confirm receiving a diagnosis of coeliac disease by a medical professional. Participants not fulfilling all the above criteria were excluded from the survey. All respondents needed to sign a consent form, which was provided in paper form at the start of the postal survey or in electronic format before the e-survey could be taken.

### Questionnaire

The questionnaire contained items on the use of health services; availability and use of prescriptions (including the costs of prescriptions and the financial impact of prescriptions being discontinued); the weekly amount spent on GFSF; amount of specific GFSF bought (bread, bread rolls, flour, bread/flour mixes, crackers/crispbreads, breakfast cereals, pasta, pizza bases, biscuits, cakes and oats); affordability of GFSF; demographics and health-related variables (see Peters et al. [26] for details).

The survey also comprised the EuroQol 5 Dimension 5 Level (EQ-5D-5L) [28] and the CDAQ [29] to assess quality of life and the Coeliac Disease Adherence Test (CDAT) to assess dietary adherence [30]. The CDAQ findings have been reported previously [26].

The EQ-5D-5L [28], a generic preference-based measure of health, includes a descriptive system of five questions on the domains of mobility, self-care, usual activities, pain, and depression/anxiety. Each question has five ordered responses that range from ‘1’ indicating no problems to ‘5’ indicating extreme problems. The instrument also includes a visual analogue scale (EQ-VAS) to measure overall health, ranging from 0 (the worst health imaginable) to 100 (the best health imaginable). Using the algorithm recommended by the National Institute for Health and Care Excellence [31, 32], EQ-5D utility scores were derived from the EQ-5D descriptive system.

The CDAT is a 7-item questionnaire that asks people with coeliac disease to self-report adherence to the gluten-free diet [30]. Each question has five response options that are summed to give a score between 7 and 35, with higher scores representing worse dietary adherence.

### Analysis

Participants were matched with information on their local prescribing policy and Index of Multiple Deprivation (IMD) scores [33]. As some participants reported not having prescriptions despite living in a prescribing area (and a small number had prescriptions despite living in a non-prescribing area), a variable of ‘self-reported prescription status’ (with three categories: ‘had prescriptions’, ‘did not have prescriptions’ and ‘had restricted prescriptions’) was computed. A ‘had restricted prescriptions’ category was added as some people reported ‘restricted prescriptions’ (meaning they could have a smaller quantity or range of GFSF on prescription) despite living in a prescribing or non-prescribing area.

The amount spent by participants each month on GFSF was calculated in two ways. Firstly, this amount was estimated by calculating the costs for the GFSF items they reported buying each month. Of several peer-reviewed publications reporting costs for the common GFSF items in the UK, the most recent costs were available in the paper by Hanci and Jeanes [15]. The costs reported in this paper were used for bread, flour, bread/flour mixes, crackers/crispbread, breakfast cereal, pasta, cakes and biscuits. The costs for pizza bases and oats were based on Fry et al. [34] and Burden et al. [16], respectively, as these were not available from Hanci and Jeanes [15]. Secondly self-reported weekly GFSF spending was used to calculate the monthly GFSF spending. All costs were adjusted for 2021 inflation rates [35].

A categorical variable using seven bands (from ‘less than £10,000’ to ‘more than £70,000’) was included in the survey and was used to calculate the mean equivalised income. First, the midpoint for each band was used to derive household income at the participant level, with the highest band (> £70,000) closed at £100,000. Then, equivalised income was calculated using the method proposed by Graville and Sutton [36] (equivalised income = household income/ √(adults + 0.5 × children)). Equivalised income could not be calculated for 21.4% of cases due to missing data on gross family income (*n* = 91, 5.4% missing and *n* = 254, 15% indicating they did not wish to answer) or household size (*n* = 53, 3.1%).

Descriptive statistics [means (SD)], chi-squared tests or analysis of variance (ANOVA) were used to examine differences in demographic and health-related variables between prescribing and non-prescribing areas and between self-reported status of receiving and not receiving prescriptions. Multivariate linear regression analyses were conducted where relevant with the estimated spending on GFSF as the outcome variable; the local area prescribing policy and affordability to buy GFSF as main explanatory variables; and demographics and health-related variables as additional explanatory variables. Sensitivity analysis was also conducted using self-reported spending on GFSF as the outcome variable and replacing local area prescribing policy with respondent self-reported prescription status as the main explanatory variable. The level of significance was set at *p* < 0.05. Data were analysed in SPSS version 29.

## Results

A total of 1653 responses (40.8%) were received for the postal survey, and 234 responses were received for the e-survey. Respondents (*n* = 151) were excluded if they were below the age limit; not living in England; did not confirm receiving a diagnosis of coeliac disease by a medical professional; or if the majority (or all) of the answers were missing. This gave a sample size of 1736 participants. Of these, 39 lived in areas that restricted prescriptions and were excluded from the data analysis, as there was no consistency in the amount and type of restrictions. Hence, the sample for analysis was 1697, with 809 participants residing in areas that provided prescriptions and 888 participants living in non-prescribing areas. The participants’ demographics and disease-related characteristics are presented in Table 1. In the non-prescribing areas, there was a significantly lower proportion of people in employment (*p* = 0.042) and a higher proportion of retired people (*p* = 0.018), a higher likelihood of living in a more deprived area (*p* < 0.001) and a lower equivalised household income (*p* = 0.001). The sample was predominantly White (99%). There was no difference between areas in the health status of participants as measured by the EQ-5D and in their adherence to a gluten-free diet as measured by the CDAT (Table 1).

**Table 1:** Demographics and health-related variables

**Demographics/Health-related variables**		**Prescribing Areas**			**Non-prescribing Areas**			***p* value**^**+**^
		**n**	**Mean**	**SD**	**n**	**Mean**	**SD**	
**Age (years) (*n* = 1659)**		794	59.4	15.8	865	60.9	15.9	0.064
**Equivalised household income**^**b**^** (*n* = 1333)**		629	24,224	15,314	704	21,601	13,864	0.001
**Time since diagnosis of coeliac disease (years) (*n* = 1657)**		795	13.88	12.97	862	13.92	12.41	0.947
**Number of comorbidities (*n* = 1697)**		809	1.93	1.55	888	1.85	1.58	0.308
**Coeliac Disease Adherence Test (CDAT) score (*n* = 1628)**		771	12.39	3.61	857	12.20	3.40	0.284
**EQ-5D Utility score (*n* = 1637)**		781	0.79	0.21	856	0.78	0.22	0.149
**EQ-5D VAS score (*n* = 1684)**		806	75.07	17.58	878	75.10	17.37	0.969
		**n**		**%**	**n**		**%**	
**Sex (*n* = 1690)**	Female	576		71.6	663		71.5	0.990
	Male	229		28.4	252		28.5	
**Marital status (*n* = 1685)**	Single	140		17.5	127		14.4	0.149
	Married or civil partnership	524		65.3	574		65.0	
	Separated, divorced or legally dissolved civil partnership	73		9.1	83		9.4	
	Widowed or survivor of civil partnership	65		8.1	99		11.2	
**Occupation** (NB. multiple responses are possible) **(*n* = 1689)**	Employment (full-time, part-time or self-employed)^a^	359		44.6	351		39.7	0.042
	Education (full- or part-time)	19		2.4	15		1.7	0.334
	Unemployed	12		1.5	9		1.0	0.381
	Permanently sick or disabled	28		3.5	31		3.5	0.975
	Retired^a^	358		44.5	444		50.2	0.018
	Looking after the home	120		14.9	134		15.2	0.885
	Other (e.g. volunteering/being a carer)	94		11.7	80		9.0	0.076
**Socioeconomic background (*n* = 1625)**	Higher & intermediate managerial	228		29.9	248		28.8	0.064
	Supervisory or clerical	268		35.1	267		31.0	
	Skilled manual	46		6.0	66		7.7	
	Semi and unskilled manual	23		3.0	42		4.9	
	State pensioners or widows	165		21.6	212		24.6	
	Casual or minimum wage earners	33		4.3	27		3.1	
**Ethnicity (*n* = 1671)**	White	796		98.9	875		99.1	0.781
	Indian	2		0.2	0		0	
	Pakistani/Bangladeshi	1		0.1	1		0.1	
	Black/African/Caribbean/Black British	2		0.2	2		0.2	
	Mixed/multiple ethnic groups	3		0.4	3		0.3	
	Other	1		0.1	2		0.2	
**Index of Multiple Deprivation quintiles**^**b**^** (*n* = 1654)**	1 – most deprived	89		11.4	67		7.7	< 0.001
	2	140		18.0	205		23.4	
	3	161		20.7	241		27.5	
	4	184		23.6	199		22.7	
	5 - least deprived	205		26.3	163		18.6	
**Affordability to buy GFSF**^**c**^** (*n* = 1636)**	Can comfortably buy GFSF I need	433		56.4	474		54.6	0.346
	Only just able to buy GFSF I need	235		30.6	293		33.8	
	Cannot always afford to buy GFSF I need	100		13.0	101		11.6	
**Self-report prescription status**^**b**^** (*n* = 1583)**	Current prescriptions	311		42.3	14		1.7	< 0.001
	Restricted prescriptions	178		24.2	32		3.8	
	No prescriptions	247		33.6	801		94.6	
**Overall impact of coeliac disease (*n* = 1678)**	No impact	148		18.4	158		18.1	0.792
	Mild impact	297		37.0	325		37.1	
	Moderate impact	250		31.1	286		32.7	
	Severe impact	84		10.5	77		8.8	
	Very severe impact	24		3.0	29		3.3	
**Availability of GFSF (*n* = 1687)**	Very easy	228		28.4	234		26.5	0.644
	Fairly easy	425		52.9	469		53.1	
	Neither easy nor difficult	91		11.3	99		11.2	
	Fairly difficult	48		6.0	68		7.7	
	Very difficult	11		1.4	14		1.6	

*GFSF* Gluten free substitute food

+*p* values for the mean difference between prescribing and non-prescribing areas

^a^Significant at the 5% level

^b^Significant at the 1% level

^c^The ‘rarely or never buy GFSF’ category removed as not relevant

### Use of prescriptions

Participants self-report of their prescription did not always match the prescription policy of the area they lived in. Of 736 participants living in prescribing areas, 247 (33.6%) reported not using prescriptions, and 178 (24.2%) reported receiving restricted prescriptions. A small percentage of participants residing in non-prescribing areas received full (*n* = 14, 1.7%) or restricted (*n* = 32, 3.8%) prescriptions. When the local area prescribing policy was compared with participants’ self-reported prescription status, participants were significantly more likely to self-report not having prescriptions in areas that had stopped prescriptions (*p* < 0.001) (Table 1).

### Access and availability of gluten-free substitute foods

There was no statistically significant difference (*p* = 0.644) in how easy or difficult participants found it to obtain GFSF between prescribing and non-prescribing areas, but 8.4% of participants (*n* = 141) found it ‘fairly difficult’ or ‘very difficult’ to buy GFSF (Table 1). Similarly, there was no statistically significant difference between prescribing and non-prescribing areas for the availability of GFSF in various locations, such as supermarkets (*p* = 0.136), health food shops (*p* = 0.570), online specialist retailers (*p* = 0.210), local corner/convenience stores (*p* = 0.258), restaurants/pubs (*p* = 0.760), cafes (*p* = 0.596), and leisure events (*p* = 0.062), with the exception of gluten-free food events (*p* < 0.001). Overall, participants indicated that it was much easier to access GFSF in supermarkets and not so much in local corner/convenience stores, while more than 50% of participants had not tried accessing online specialist retailers and gluten-free food events. Of the 44% who had attended gluten-free food events, participants in non-prescribing areas indicated less availability of GFSF than participants in prescribing areas.

### Amount of gluten-free substitute foods bought and estimated spending

Participants in non-prescribing areas purchased all types of GFSF items, apart from biscuits and cakes, in statistically significantly higher quantities and thereby spent an additional £11.32/month on GFSF items than participants in prescribing areas (*p* < 0.001) (Table 2). Similarly, participants who self-reported that they did not get prescriptions (irrespective of the prescription policy of their area) bought higher quantities of most GFSF items (excluding bread/flour mixes, biscuits and cakes) and thereby spent an additional £11.89 per month than participants self-reporting receiving prescriptions (both getting full or restricted prescriptions) (*p* < 0.001) (Table 3). This analysis was repeated after removing the participants whose self-reported prescription status did not match the prescription policy of the area (*n* = 247, 33.6% in prescribing areas; *n* = 46, 5.5% in non-prescribing areas). The difference in the amount spent on GFSF between prescribing and non-prescribing areas increased to £14.09/month (*p* < 0.001).

**Table 2:** Number of packs of GFSF bought in a typical month and its estimated cost (£) by prescription policy

**GFSF item (weight per pack in g)**	**Prescribing areas**			**Non-prescribing areas**			**Mean difference in number of packs bought between non-prescribing and prescribing areas (95% CI)**	**Mean difference in cost (£) between non-prescribing and prescribing areas (95% CI)**	***p*** **value**
	**N**	**Number of packs bought**	**Cost (£)**	**N**	**Number of packs bought**	**Cost (£)**			
		**Mean ± SD**	**Mean ± SD**		**Mean ± SD**	**Mean ± SD**			
**Bread (480 g)**	794	2.40 ± 2.56	7.96 ± 8.51	881	4.16 ± 3.19	13.82 ± 10.60	1.77 (1.49 to 2.05)	5.86 (4.93 to 6.79)	< 0.001
**Bread roll (175 g)**	798	1.12 ± 1.99	1.90 ± 3.38	882	1.42 ± 2.04	2.41 ± 3.47	0.30 (0.10 to 0.49)	0.50 (0.18 to 0.83)	0.003
**Flour (500 g)**	798	0.49 ± 0.77	0.47 ± 0.74	882	0.83 ± 0.96	0.81 ± 0.93	0.34 (0.26 to 0.43)	0.33 (0.25 to 0.41)	< 0.001
**Bread or flour mixes (500 g)**	798	0.16 ± 0.70	0.16 ± 0.68	882	0.37 ± 1.95	0.36 ± 1.89	0.21 (0.07 to 0.35)	0.20 (0.06 to 0.34)	0.004
**Crackers or crispbreads (200 g)**	798	1.15 ± 1.64	3.97 ± 5.66	881	1.48 ± 1.74	5.11 ± 6.03	0.33 (0.17 to 0.49)	1.14 (0.58 to 1.70)	< 0.001
**Breakfast cereal (300 g)**	797	1.72 ± 1.75	4.72 ± 4.82	881	2.00 ± 1.91	5.49 ± 5.26	0.28 (0.11 to 0.46)	0.78 (0.29 to 1.26)	0.002
**Pasta (250 g)**	798	1.07 ± 1.46	1.18 ± 1.62	882	1.35 ± 1.51	1.50 ± 1.67	0.28 (0.14 to 0.43)	0.32 (0.16 to 0.47)	< 0.001
**Pizza bases (200 g)**	798	0.27 ± 0.72	0.90 ± 2.38	880	0.48 ± 1.29	1.59 ± 4.25	0.21 (0.11 to 0.31)	0.69 (0.36 to 1.03)	< 0.001
**Biscuits (150 g)**	798	2.35 ± 2.60	4.96 ± 5.49	882	2.54 ± 2.67	5.35 ± 5.63	0.18 (-0.07 to 0.44)	0.39 (-0.14 to 0.92)	0.153
**Cakes (200 g)**	797	1.27 ± 1.81	2.27 ± 3.24	880	1.23 ± 1.72	2.21 ± 3.09	-0.04 (-0.21 to 0.13)	-0.07 (-0.37 to 0.24)	0.665
**Oats (500 g)**	797	0.61 ± 1.09	2.27 ± 4.02	882	0.93 ± 1.82	3.43 ± 6.75	0.31 (0.17 to 0.46)	1.16 (0.62 to 1.70)	< 0.001
**Estimated amount spent on GFSF in a typical month**	790	-	30.81 ± 18.78	876	-	42.13 ± 23.50	-	11.32 (9.27 to 13.38)	< 0.001

Cost of items per pack: Bread (480g): £3.32; Bread rolls (175g): £1.70; Flour (500g): £0.97; Bread/flour mixes (500g): £0.97; Crackers/Crispbread (200g): £3.46; Breakfast cereal (300g): £2.75; Pasta (250g): £1.11; Pizza bases (200g): £3.31; Biscuits (150g): £2.11; Cakes (200g): £1.79; Oats (500g): £3.70

**Table 3:** Number of packs of GFSF bought in a typical month and its estimated cost (£) by self-reported prescription status (irrespective of the local area prescribing policy)

**GFSF item (weight per pack in g)**	**Full prescription or restricted prescription**			**No prescription**			**Mean difference in number of packs bought between no prescription group and prescription group (95% CI)**	**Mean difference in cost (£) between no prescription group and prescription group (95% CI)**	***p*** **value**
	**N**	**Number of packs bought**	**Cost (£)**	**N**	**Number of packs bought**	**Cost (£)**			
		**Mean ± SD**	**Mean ± SD**		**Mean ± SD**	**Mean ± SD**			
**Bread (480 g)**	527	2.11 ± 2.43	6.99 ± 8.06	1041	3.97 ± 3.13	13.19 ± 10.38	1.87 (1.56 to 2.17)	6.20 (5.19 to 7.21)	< 0.001
**Bread roll (175 g)**	530	1.03 ± 1.82	1.75 ± 3.10	1042	1.41 ± 2.13	2.40 ± 3.62	0.38 (0.17 to 0.57)	0.65 (0.29 to 1.01)	< 0.001
**Flour (500 g)**	530	0.47 ± 0.78	0.45 ± 0.76	1042	0.77 ± 0.911	0.75 ± 0.88	0.30 (0.21 to 0.39)	0.29 (0.21 to 0.38)	< 0.001
**Bread or flour mixes (500 g)**	530	0.18 ± 0.65	0.17 ± 0.63	1042	0.32 ± 1.83	0.31 ± 1.77	0.15 (-0.02 to 0.31)	0.14 (-0.01 to 0.30)	0.075
**Crackers or crispbreads (200 g)**	530	1.05 ± 1.46	3.62 ± 5.03	1042	1.48 ± 1.77	5.13 ± 6.14	0.44 (0.26 to 0.61)	1.50 (0.90 to 2.11)	< 0.001
**Breakfast cereal (300 g)**	529	1.69 ± 1.71	4.65 ± 4.70	1041	1.98 ± 1.94	5.45 ± 5.33	0.29 (0.09 to 0.48)	0.79 (0.26 to 1.33)	0.004
**Pasta (250 g)**	530	0.88 ± 1.44	0.98 ± 1.60	1042	1.40 ± 1.49	1.56 ± 1.66	0.52 (0.37 to 0.68)	0.58 (0.41 to 0.75)	< 0.001
**Pizza bases (200 g)**	530	0.28 ± 0.70	0.93 ± 2.31	1041	0.43 ± 1.22	1.43 ± 4.04	0.15 (0.04 to 0.26)	0.49 (0.12 to 0.86)	0.009
**Biscuits (150 g)**	530	2.44 ± 2.63	5.14 ± 5.54	1042	2.45 ± 2.64	5.16 ± 5.56	0.01 (-0.27 to 0.29)	0.02 (-0.56 to 0.60)	0.938
**Cakes (200 g)**	530	1.28 ± 1.83	2.29 ± 3.27	1039	1.25 ± 1.76	2.24 ± 3.15	-0.03 (-0.21 to 0.16)	-0.05 (-0.38 to 0.29)	0.784
**Oats (500 g)**	530	0.54 ± 0.95	1.99 ± 3.52	1041	0.86 ± 1.30	3.19 ± 4.81	0.33 (0.2 to 0.45)	1.20 (0.74 to 1.67)	< 0.001
**Estimated amount spent on GFSF in a typical month**	526	-	28.99 ± 17.88	1035		40.87 ± 21.85	-	11.89 (9.73 to 14.05)	< 0.001

Cost of items per pack: Bread (480g): £3.32; Bread rolls (175g): £1.70; Flour (500g): £0.97; Bread/flour mixes (500g): £0.97; Crackers/Crispbread (200g): £3.46; Breakfast cereal (300g): £2.75; Pasta (250g): £1.11; Pizza bases (200g): £3.31; Biscuits (150g): £2.11; Cakes (200g): £1.79; Oats (500g): £3.70

### Self-reported spending on gluten-free substitute foods

Self-reported spending was similar to the estimated spending, with participants in the non-prescribing areas self-reporting spending more on GFSF in a typical month than their counterparts in prescribing areas; however, this difference was lower (£5.92) and not statistically significant (*p* = 0.058) (Table 4). Interestingly, self-reported spending was nearly two times higher than the estimated spending in both the prescribing and non-prescribing areas (*p* < 0.001) (Table 4). The findings were similar when self-reported prescription status was taken into consideration, except that the difference in self-reported spending between participants receiving and not receiving prescriptions was higher at £14.79 and statistically significant (*p* < 0.001) (Table 4).

**Table 4:** Comparison of the estimated spending and self-reported spending (£) on GFSF in a typical month by the local area prescription policy and self-reported prescription status

**Spending on GFSF**	**Prescribing areas**		**Non-prescribing areas**		**Mean difference (95% CI) in amount spent between prescribing and non-prescribing areas**	***p* value**
	**N**	**Mean ± SD**	**N**	**Mean ± SD**		
**Self-reported spending on GFSF per month**	766	75.50 ± 64.56	842	81.42 ± 60.70	5.92 (-0.20 to 12.05)	0.058
**Estimated spending on GFSF per month**	790	30.81 ± 18.78	876	42.13 ± 23.50	11.32 (9.27 to 13.38)	< 0.001
**Mean difference (95% CI) between the self-reported and estimated spending on GFSF in a typical month**		44.69 (40.23 to 48.83)		39.29 (35.65 to 43.33)	-	-
***p*** **value**		< 0.001		< 0.001		
	**Self-reported prescriptions (full & restricted)**		**Self-reported no prescriptions**		**Mean difference (95% CI) in amount spent based on self-reported prescription status**	***p* value**
	**N**	**Mean ± SD**	**N**	**Mean ± SD**		
**Self-reported spending on GFSF per month**	507	68.36 ± 56.76	996	83.15 ± 64.25	14.79 (8.17 to 21.40)	< 0.001
**Estimated spending on GFSF per month**	526	28.99 ± 17.88	1035	40.87 ± 21.85	11.89 (9.73 to 14.05)	< 0.001
**Mean difference (95% CI) between the self-reported and estimated spending on GFSF in a typical month**		39.37 (35.05 to 44.08)		42.28 (38.68 to 46.18)	-	-
***p*** **value**		< 0.001		< 0.001		

### Affordability to buy gluten-free substitute foods

The participants’ views on the affordability of GFSF did not differ between participants based on the local area policy (*p* = 0.346) (Table 1) or by self-reported prescription status (*p* = 0.755), with more than 50% of participants indicating that they can comfortably afford to buy the GFSF they need. In both the prescribing and non-prescribing areas, participants who reported being comfortably able to buy GFSF had a statistically significant higher mean equivalised income than those who were only just able to afford GFSF and those who could not always afford it (*p* < 0.001) (Table 5). However, participants’ views on the affordability of GFSF did not influence their spending on GFSF across both prescribing and non-prescribing areas. For example, participants in non-prescribing areas who were only just able to afford to buy GFSF appeared to spend more on GFSF than participants who were able to comfortably buy GFSF (Table 5). The findings were similar for participants who self-reported not receiving prescriptions (Table 5).

**Table 5:** Affordability to buy GFSF compared with the amount spent on GFSF and the equivalised income based on local area prescription policy and self-reported prescription status

**Affordability to buy GFSF**	**Estimated spending on GFSF per month (£)**				**Equivalised income (£)**			
	***Prescribing areas (n* = *757)***		***Non-prescribing areas (n* = *858)***		***Prescribing areas (n* = *605)***		***Non-prescribing areas (n* = *691)***	
	**N**	**Mean ± SD**	**N**	**Mean ± SD**	**N**	**Mean ± SD**	**N**	**Mean ± SD**
Can comfortably buy GFSF I need	430	30.95 ± 18.38	469	41.01 ± 23.26	335	30,640 ± 15,543	367	26,848 ± 14,791
Only just able to buy GFSF I need	229	31.20 ± 18.07	289	45.68 ± 24.26	190	17,516 ± 12,098	237	16,270 ± 9,656
Cannot always afford to buy GFSF I need	98	34.91 ± 21.61	100	40.12 ± 21.18	80	15,524 ± 9,055	87	13,321 ± 7,554
*p* value		0.16		< 0.001		< 0.001		< 0.001
	***Self-report prescriptions (full & restricted) (n* = *502)***		***Self-reported no prescriptions (n* = *1013)***		***Self-reported prescriptions (full & restricted) (n* = *405)***		***Self-reported no prescriptions (n* = *815)***	
	**N**	**Mean ± SD**	**N**	**Mean ± SD**	**N**	**Mean ± SD**	**N**	**Mean ± SD**
Can comfortably buy GFSF I need	279	28.45 ± 17.68	570	39.79 ± 20.04	219	27,552 ± 14,138	445	29,401 ± 15,823
Only just able to buy GFSF I need	157	29.56 ± 16.79	322	44.69 ± 24.30	134	16,876 ± 11,763	263	17,289 ± 10,536
Cannot always afford to buy GFSF I need	66	34.47 ± 20.48	121	39.38 ± 21.47	52	14,229 ± 8,004	107	14,585 ± 8,710
*p* value		0.048		0.03		< 0.001		< 0.001

### Regression analysis

Since the estimated spending on GFSF differed significantly between participants based on local area prescription policy, this variable was examined further using regression analysis. The regression model was statistically significant (R^2^_adj_ 0.11, *p* < 0.001) (*n* = 1074), with the estimated monthly spending on GFSF higher in participants living in non-prescribing areas (*p* < 0.001); in participants who were just able to afford to buy GFSF (*p* < 0.001); in males (*p* < 0.001); and where there were additional household members with coeliac disease (*p* < 0.001) (Table 6 and Supplementary file 1 (S Table 1)).

**Table 6:** Significant variables in regression analysis

Regression variables	Model significance	Significant explanatory variables
Estimated spending (outcome) and local area prescription policy (main explanatory variable) (*n* = 1074)	*R*^2^_adj_ 0.11, *p* < 0.001	Spending on GFSF was higher (i) in participants living in non-prescribing areas (*p* < 0.001) (ii) in participants who were just able to afford to buy GFSF (*p* < 0.001) (iii) in males (*p* < 0.001) (iv) where there were additional household members with coeliac disease (*p* < 0.001)
Estimated spending (outcome) and self-report prescription status (main explanatory variable) (*n* = 1074)	*R*^2^_adj_ 0.11, *p* < 0.001	Spending on GFSF was higher (i) in participants living in non-prescribing areas (*p* < 0.001) (ii) in participants who were just able to afford to buy GFSF (*p* < 0.001) (iii) in males (*p* < 0.001) (iv) where there were additional household members with coeliac disease (*p* < 0.001) Spending on GFSF was lower (i) in participants who are separated/divorced (*p* = 0.028)
Self-reported spending (outcome) and local area prescription policy (main explanatory variable) (*n* = 1074)	*R*^2^_adj_ 0.10, *p* < 0.001	Spending on GFSF was higher (i) in participants living in non-prescribing areas (*p* < 0.001) (ii) in participants who were just able to afford to buy GFSF (*p* < 0.001) (iii) in males (*p* < 0.001) (iv) where there were additional household members with coeliac disease (*p* = 0.003) Spending on GFSF was lower (i) as age increase (*p* < 0.001) (ii) in socioeconomic domain of supervisory or clerical (*p* = 0.017) and skilled manual work (*p* = 0.042)
Self-reported spending (outcome) and self-reported prescription status (main explanatory variable) (*n* = 1074)	*R*^2^_adj_ 0.10, *p* < 0.001	Spending on GFSF was higher (i) in participants living in non-prescribing areas (*p* < 0.001) (ii) in participants who were just able to afford to buy GFSF (*p* < 0.001) (iii) in males (*p* < 0.001) (iv) where there were additional household members with coeliac disease (*p* = 0.002) Spending on GFSF was lower (i) as age increase (*p* < 0.001) (ii) in socioeconomic domain of supervisory or clerical (*p* = 0.016) and skilled manual work (*p* = 0.046)
Estimated spending (outcome) and local area prescription policy (main explanatory variable). Sample restricted to participants whose self-report prescription status matched with the local area prescription policy (*n* = 784)	*R*^2^_adj_ 0.16, *p* < 0.001	Spending on GFSF was higher (i) in participants living in non-prescribing areas (*p* < 0.001) (ii) in participants who were just able to afford to buy GFSF (*p* < 0.001) (iii) in males (*p* < 0.001) (iv) where there were additional household members with coeliac disease (*p* < 0.001)

Sensitivity analyses were undertaken by replacing ‘local area prescription policy’ with ‘self-reported prescription status’ (Supplementary file 1 (S Tables 2 and 4)) as the main explanatory variable and ‘estimated spending on GFSF’ with ‘self-reported spending on GFSF’ (Supplementary file 1 (S Tables 3 and 4)) as the outcome, using the same sample of participants for comparability. The significance of the sensitivity analysis models (Supplementary file 1 (S Tables 2 to 4)) was similar to that of the main model specification (Supplementary file 1 (S Table 1)). As summarised in Table 6, all models were statistically significant, and there was little change in the significance of the explanatory variables across model specifications (Table 6 and Supplementary file 1 (S Tables 1 to 4)). In addition, the self-reported spending on GFSF was higher for participants who were not able to afford GFSF, lower as age increased, and in participants within the socioeconomic domain of supervisory or clerical and skilled manual work (Table 6 and Supplementary file 1 (S Tables 1 to 4)). When the main model specification (Supplementary file 1 (S Table 1)) was estimated after restricting the sample to only participants whose self-reported prescription status matched the local area prescription policy, the significance of the model improved marginally; however, the significant variables remained the same as in the original model [*R*^2^_adj_ 0.16, *p* < 0.001 (*n* = 784)] (Tables 5 and 6).

## Discussion

This study showed that although there was no difference in the ease of access and availability of GFSF between prescribing and non-prescribing areas, participants in non-prescribing areas were buying more GFSF out of pocket and thereby spending more on GFSF as opposed to those in prescribing areas, who are more likely to get some of their GFSF on prescription. Specifically, households with multiple members with coeliac disease and people who are just able/not able to afford GFSF appeared to be spending more on GFSF. In line with these findings, Vriesekoop et al. [37] observed that the availability of GFSF increased from 2015 to 2019, including GFSF stocking in budget supermarkets. However, the quality and pricing of GFSF continues to be a major issue not only in the UK but also across the world [14, 17, 37, 38]. A recent study conducted by Coeliac UK demonstrated that a gluten-free food shopping basket (based on minimum income standard and containing around 70 items each) could be 20 percent more expensive than a standard food shopping basket [38]. Another study estimated a mean increase of £861 per person with coeliac disease per year in food costs alone [39]. Therefore, the difference noted in GFSF spending in the current study (£10 to £15 per month) between prescribing and non-prescribing areas could be a significant, meaningful difference. It is likely that the estimated spending could be an underestimation, as it was not possible to calculate costs for food items reported under ‘other’ in the questionnaire (e.g., ready meals); therefore, the difference in spending could be even larger.

Regression analysis of the current study indicated that households with multiple members with coeliac disease were spending more on GFSF. Given the increase in the overall incidence of coeliac disease [40] and the increased risk of coeliac disease for first-degree family members of someone with coeliac disease (due to genetic links) [1], the possibility of having households with multiple members with coeliac disease is likely to increase and become more common. This may lead to a significant economic strain for a higher proportion of the population.

Regression analysis also indicated that people who are just able/not able to afford GFSF were spending more on GFSF. Moreover, affordability to buy GFSF was dependent on the equivalised income, with people on lower income not always able to afford to buy GFSF. According to the UK Government’s family food survey, households in the poorest decile spend only around £21.95 per week on food and non-alcoholic drinks [41]. For such families, the added cost of £10 to £15 per month for buying GFSF may be hugely significant and, in some cases, unaffordable too. Unsurprisingly, this subset of population, along with people who are less able to travel to large shops (e.g., people with disabilities), seemed to be the most impacted by prescription withdrawal in relation to quality of life as well [26]. The qualitative study on the affordability and obtainability of GFSF based on a sub-set of this sample identified these groups as the most impacted [27]. Poverty is more likely in certain groups of the population (e.g., single parents, those with disabilities, some ethnic minority groups) [42]. These factors combined with the current economic crisis and increased food inflation in the UK could be additionally damaging for this subset of the population [38].

Since the study was completed, two important and relevant policy changes have been made in England. Firstly, in 2018, the UK Government’s Department of Health and Social Care recommended limiting prescriptions for coeliac disease only to bread and flour mixes in England based on a public consultation in 2017 [43]. However, it was left to the local area government bodies to uptake/reject this recommendation, resulting in continued variation in the prescription policy across areas. Secondly, the local area government bodies (CCGs), which were the statutory bodies responsible for planning and commissioning services in England at the time of the study, have been replaced by Integrated Care Boards (ICB) since July 2022 [44]. While ICBs are required to have unified policies across the areas under their remit, it is not clear how prescription variations within the areas will be dealt with and how this will impact the population. The current study has demonstrated that prescription withdrawal has significant financial implications, and therefore, it is crucial that newly formed ICBs and wider policy makers take this into consideration when making any further changes to prescription provisions for people with coeliac disease.

## Limitations

The limitations of the study need to be acknowledged. Although the response rate of 40.8% is in line with previous similar surveys [5, 29], it is not known if the spending of the non-responders would have been similar or different. Although several factors such as geographical location and level of deprivation were considered and random sampling was used in local areas with large membership, the final sample was predominantly White and the reasons for this could not be explored in the current study. This sample is not reflective of the increasing non-white population in the UK [45], and at least some of the ethnic minority groups are socially disadvantaged [42]. Given that dietary adherence appears to be different between Caucasians and South Asians [25] and that some minority ethnic groups are in the lowest income groups in the country [46], including ethnic minority groups in future studies is crucial. The cost of food items upon which estimates were based varied between published papers, with no consistency in the methods used to calculate costs (median, average, lowest price of an item). Therefore, there may be an under- or overestimation of the cost of individual food items, and therefore, the estimated spending on GFSF may be an under- or overestimation. In addition, the estimated spending may also be an underestimate, as it was not possible to calculate costs for items mentioned under ‘others’ (e.g., ready meals). Furthermore, the potential cost of prescriptions for participants was not taken into account in the analysis.

## Implications

- People with coeliac disease buy and spend more on GFSF in areas that did not provide prescriptions as per the national prescribing guidelines at the time.
- People with coeliac disease with a lower annual equivalised income appear to not always be able to afford to buy GFSF.
- Future prescription changes in coeliac disease should consider these financial implications.

## Conclusions

In conclusion, this study shows that people living in non-prescribing areas spent (statistically) significantly more money on GFSF than people living in prescribing areas. Whilst for many people this may be affordable, this increase in spending may be difficult to afford for more vulnerable groups (e.g., those with lower incomes). Further research is needed on how more diverse and potentially vulnerable groups are affected and to what extent affordability of GFSF contributes to health inequalities. Given the recent formation of the integrated care system, any changes to prescription policy should consider the impact on the population, especially lower income households.

### Supplementary Information

**Additional file 1:** Regression analysis results.

## Data Availability

The data that support the findings of this study are available from the corresponding author upon reasonable request.

## Abbreviations

GFSF: Gluten-free substitute foods
NHS: National Health Service
CCG: Clinical Commissioning Group
CDAQ: Coeliac Disease Assessment Questionnaire
CDAT: Coeliac Disease Adherence Test
ICB: Integrated Care Board
