# Supplementary material for: The financial impact on people with coeliac disease of withdrawing gluten-free food from prescriptions in England: findings from a cross-sectional survey
Source: BMC Health Serv Res. 2024 Jan 30;24:146. doi: 10.1186/s12913-024-10600-4 (PMC10826048; doi:10.1186/s12913-024-10600-4)
Supplement: Supplementary file 1 — Additional file 1: Regression analysis results. [file 12913_2024_10600_MOESM1_ESM.docx]

## Supplementary file 1

**S Table 1: Regression analysis for estimated amount spent on GFSF in a month (outcome variable) and local area prescription policy (main explanatory variable) (R^2^_adj_ 0.11, p<0.001) (n=1074)**

| **Variables** | **Unstandardized coefficient** | | **Standardized Coefficients β** | **t** | **95% CI** | | **p** |
| --- | --- | --- | --- | --- | --- | --- | --- |
|  | **β** | **Standard error** |  |  | **Lower** | **Upper** |  |
| Local area prescription policy | 9.96 | 1.26 | 0.23 | 7.89 | 7.48 | 12.43 | <.001 |
| Affordability to buy GFSF |  |  |  |  |  |  |  |
| Can comfortably buy GFSF | Ref | - | - | - | - | - | - |
| Can just afford to buy GFSF | 5.78 | 1.57 | 0.13 | 3.68 | 2.70 | 8.85 | <.001 |
| Cannot always afford to buy GFSF | 2.47 | 2.26 | 0.04 | 1.10 | -1.96 | 6.90 | 0.274 |
| Sex | 9.15 | 1.52 | 0.20 | 6.02 | 6.17 | 12.14 | <.001 |
| Age | 0.03 | 0.07 | 0.02 | 0.36 | -0.11 | 0.16 | 0.72 |
| Occupation |  |  |  |  |  |  |  |
| Employed (full time, part time and self- employed)’ | Ref | - | - | - | - | - | - |
| Unemployed | 6.52 | 7.86 | 0.03 | 0.83 | -8.90 | 21.94 | 0.407 |
| Permanently sick or disabled | 3.76 | 3.62 | 0.03 | 1.04 | -3.34 | 10.85 | 0.299 |
| Fully retired from work | -3.09 | 1.87 | -0.07 | -1.66 | -6.76 | 0.57 | 0.098 |
| Looking after the home | 0.56 | 1.90 | 0.01 | 0.29 | -3.17 | 4.28 | 0.769 |
| ‘Other’ employment | -0.45 | 2.17 | -0.01 | -0.21 | -4.71 | 3.82 | 0.838 |
| Full or part-time education | -2.17 | 4.70 | -0.02 | -0.46 | -11.39 | 7.06 | 0.645 |
| Marital status |  |  |  |  |  |  |  |
| Married | Ref | - | - | - | - | - | - |
| Single | -2.75 | 2.01 | -0.05 | -1.37 | -6.69 | 1.19 | 0.172 |
| Separated, divorced or legally dissolved civil partnership | -3.78 | 2.15 | -0.05 | -1.76 | -8.01 | 0.44 | 0.079 |
| Widowed or surviving partner of civil partnership | -0.97 | 2.51 | -0.01 | -0.39 | -5.89 | 3.94 | 0.698 |
| Socioeconomic background |  |  |  |  |  |  |  |
| Higher & intermediate managerial, administrative, professional occupations | Ref | - | - | - | - | - | - |
| Supervisory or clerical, junior managerial, administrative or professional | -2.65 | 1.73 | -0.06 | -1.53 | -6.05 | 0.75 | 0.126 |
| Skilled manual | -4.13 | 2.91 | -0.05 | -1.42 | -9.83 | 1.58 | 0.156 |
| Semi or unskilled manual | 1.77 | 3.64 | 0.02 | 0.49 | -5.37 | 8.91 | 0.627 |
| State pensioners or widows (no other earner) | -2.19 | 2.30 | -0.04 | -0.95 | -6.70 | 2.33 | 0.342 |
| Casual or minimum earners | -5.28 | 3.76 | -0.05 | -1.40 | -12.66 | 2.10 | 0.161 |
| Educational level |  |  |  |  |  |  |  |
| University undergraduate or postgraduate degree) | Ref | - | - | - | - | - | - |
| Secondary school | 1.49 | 1.99 | 0.03 | 0.75 | -2.42 | 5.40 | 0.455 |
| College | 0.63 | 1.76 | 0.01 | 0.36 | -2.82 | 4.08 | 0.720 |
| ‘Other’ | 1.10 | 2.42 | 0.02 | 0.46 | -3.64 | 5.85 | 0.648 |
| Ethnicity | 0.99 | 6.20 | 0.01 | 0.16 | -11.17 | 13.15 | 0.873 |
| Equivalized income | 0.00 | 0.00 | 0.01 | 0.27 | 0.00 | 0.00 | 0.790 |
| Number of additional household members with coeliac disease | 8.03 | 1.90 | 0.12 | 4.23 | 4.31 | 11.75 | <.001 |
| Index of Multiple Deprivation Quintiles | -0.16 | 0.52 | -0.01 | -0.30 | -1.18 | 0.87 | 0.767 |
| Time since diagnosis of coeliac disease | -0.05 | 0.05 | -0.03 | -0.93 | -0.15 | 0.05 | 0.350 |
| Total number of comorbidities | -0.10 | 0.43 | -0.01 | -0.24 | -0.95 | 0.74 | 0.811 |
| (Constant) | 28.07 | 7.75 |  | 3.62 | 12.87 | 43.27 | <.001 |

**S Table 2: Regression analysis for estimated amount spent on GFSF in a month (outcome variable) and self-reported prescription status (main explanatory variable) (R^2^_adj_ 0.13, p<0.001) (n=1074)**

| **Variables** | **Unstandardized coefficient** | | **Standardized Coefficients β** | **t** | **95% CI** | | **p** |
| --- | --- | --- | --- | --- | --- | --- | --- |
|  | **β** | **Standard error** |  |  | **Lower** | **Upper** |  |
| Self-report prescription status | 12.87 | 1.33 | 0.28 | 9.68 | 10.26 | 15.48 | <.001 |
| Affordability to buy GFSF |  |  |  |  |  |  |  |
| Can comfortably buy GFSF | Ref | - | - | - | - | - | - |
| Can just afford to buy GFSF | 5.83 | 1.55 | 0.13 | 3.77 | 2.79 | 8.86 | <.001 |
| Cannot always afford to buy GFSF | 2.18 | 2.23 | 0.03 | 0.98 | -2.19 | 6.54 | 0.328 |
| Sex | 9.65 | 1.50 | 0.21 | 6.42 | 6.70 | 12.59 | <.001 |
| Age | 0.07 | 0.07 | 0.05 | 1.04 | -0.06 | 0.21 | 0.298 |
| Occupation |  |  |  |  |  |  |  |
| Employed (full time, part time and self- employed)’ | Ref | - | - | - | - | - | - |
| Unemployed | 3.59 | 7.75 | 0.01 | 0.46 | -11.62 | 18.80 | 0.643 |
| Permanently sick or disabled | 4.22 | 3.57 | 0.04 | 1.18 | -2.77 | 11.22 | 0.237 |
| Fully retired from work | -2.79 | 1.84 | -0.07 | -1.52 | -6.41 | 0.82 | 0.130 |
| Looking after the home | 0.62 | 1.87 | 0.01 | 0.33 | -3.05 | 4.29 | 0.741 |
| ‘Other’ employment | -0.55 | 2.14 | -0.01 | -0.26 | -4.76 | 3.66 | 0.798 |
| Full or part-time education | -1.53 | 4.64 | -0.01 | -0.33 | -10.62 | 7.57 | 0.742 |
| Marital status |  |  |  |  |  |  |  |
| Married | Ref | - | - | - | - | - | - |
| Single | -3.16 | 1.98 | -0.05 | -1.60 | -7.05 | 0.72 | 0.110 |
| Separated, divorced or legally dissolved civil partnership | -4.67 | 2.13 | -0.07 | -2.20 | -8.84 | -0.50 | 0.028 |
| Widowed or surviving partner of civil partnership | -2.06 | 2.47 | -0.03 | -0.83 | -6.92 | 2.80 | 0.405 |
| Socioeconomic background |  |  |  |  |  |  |  |
| Higher & intermediate managerial, administrative, professional occupations | Ref | - | - | - | - | - | - |
| Supervisory or clerical, junior managerial, administrative or professional | -2.91 | 1.71 | -0.07 | -1.70 | -6.25 | 0.44 | 0.089 |
| Skilled manual | -3.53 | 2.87 | -0.04 | -1.23 | -9.15 | 2.09 | 0.218 |
| Semi or unskilled manual | 1.47 | 3.59 | 0.01 | 0.41 | -5.57 | 8.51 | 0.682 |
| State pensioners or widows (no other earner) | -1.97 | 2.27 | -0.04 | -0.87 | -6.42 | 2.49 | 0.386 |
| Casual or minimum earners | -4.20 | 3.72 | -0.04 | -1.13 | -11.49 | 3.09 | 0.259 |
| Educational level |  |  |  |  |  |  |  |
| University undergraduate or postgraduate degree) | Ref | - | - | - | - | - | - |
| Secondary school | 1.36 | 1.96 | 0.03 | 0.69 | -2.50 | 5.21 | 0.490 |
| College | 0.35 | 1.73 | 0.01 | 0.20 | -3.05 | 3.75 | 0.840 |
| ‘Other’ | 0.37 | 2.39 | 0.01 | 0.15 | -4.32 | 5.05 | 0.878 |
| Ethnicity | -0.26 | 6.11 | 0.00 | -0.04 | -12.25 | 11.74 | 0.966 |
| Equivalized income | 0.00 | 0.00 | -0.01 | -0.30 | 0.00 | 0.00 | 0.766 |
| Number of additional household members with coeliac disease | 8.09 | 1.87 | 0.13 | 4.32 | 4.42 | 11.76 | <.001 |
| Index of Multiple Deprivation Quintiles | -0.17 | 0.52 | -0.01 | -0.33 | -1.19 | 0.84 | 0.740 |
| Time since diagnosis of coeliac disease | -0.03 | 0.05 | -0.02 | -0.60 | -0.13 | 0.07 | 0.551 |
| Total number of comorbidities | -0.02 | 0.43 | 0.00 | -0.05 | -0.85 | 0.82 | 0.964 |
| (Constant) | 23.91 | 7.68 |  | 3.11 | 8.85 | 38.97 | 0.002 |

**S Table 3: Regression analysis for self-reported spending on GFSF in a month (outcome variable) and local area prescription policy (main explanatory variable) (R^2^_adj_ 0.10, p<0.001) (n=1074)**

| **Variables** | **Unstandardized coefficient** | | **Standardized Coefficients β** | **t** | **95% CI** | | **p** |
| --- | --- | --- | --- | --- | --- | --- | --- |
|  | **β** | **Standard error** |  |  | **Lower** | **Upper** |  |
| Local area prescription policy | 8.57 | 3.76 | 0.07 | 2.28 | 1.20 | 15.94 | 0.023 |
| Affordability to buy GFSF |  |  |  |  |  |  |  |
| Can comfortably buy GFSF | Ref | - | - | - | - | - | - |
| Can just afford to buy GFSF | 19.21 | 4.67 | 0.14 | 4.12 | 10.05 | 28.37 | <.001 |
| Cannot always afford to buy GFSF | 23.76 | 6.72 | 0.13 | 3.54 | 10.58 | 36.94 | <.001 |
| Sex | 25.44 | 4.53 | 0.18 | 5.62 | 16.55 | 34.32 | <.001 |
| Age | -0.73 | 0.21 | -0.18 | -3.51 | -1.13 | -0.32 | <.001 |
| Occupation |  |  |  |  |  |  |  |
| Employed (full time, part time and self- employed)’ | Ref | - | - | - | - | - | - |
| Unemployed | 45.31 | 23.40 | 0.06 | 1.94 | -0.60 | 91.22 | 0.053 |
| Permanently sick or disabled | 6.64 | 10.76 | 0.02 | 0.62 | -14.48 | 27.76 | 0.537 |
| Fully retired from work | -0.72 | 5.56 | -0.01 | -0.13 | -11.64 | 10.20 | 0.897 |
| Looking after the home | 0.13 | 5.65 | 0.00 | 0.02 | -10.96 | 11.22 | 0.982 |
| ‘Other’ employment | -10.28 | 6.47 | -0.05 | -1.59 | -22.98 | 2.42 | 0.112 |
| Full or part-time education | -23.35 | 13.99 | -0.05 | -1.67 | -50.80 | 4.11 | 0.096 |
| Marital status |  |  |  |  |  |  |  |
| Married | Ref | - | - | - | - | - | - |
| Single | -1.47 | 5.98 | -0.01 | -0.25 | -13.20 | 10.26 | 0.806 |
| Separated, divorced or legally dissolved civil partnership | -7.48 | 6.41 | -0.04 | -1.17 | -20.07 | 5.10 | 0.243 |
| Widowed or surviving partner of civil partnership | -5.45 | 7.46 | -0.02 | -0.73 | -20.08 | 9.18 | 0.465 |
| Socioeconomic background |  |  |  |  |  |  |  |
| Higher & intermediate managerial, administrative, professional occupations | Ref | - | - | - | - | - | - |
| Supervisory or clerical, junior managerial, administrative or professional | -12.38 | 5.16 | -0.09 | -2.40 | -22.50 | -2.26 | 0.017 |
| Skilled manual | -17.65 | 8.66 | -0.07 | -2.04 | -34.64 | -0.65 | 0.042 |
| Semi or unskilled manual | -17.14 | 10.83 | -0.05 | -1.58 | -38.39 | 4.10 | 0.114 |
| State pensioners or widows (no other earner) | -2.26 | 6.85 | -0.01 | -0.33 | -15.70 | 11.18 | 0.742 |
| Casual or minimum earners | -13.92 | 11.20 | -0.04 | -1.24 | -35.90 | 8.06 | 0.214 |
| Educational level |  |  |  |  |  |  |  |
| University undergraduate or postgraduate degree) | Ref | - | - | - | - | - | - |
| Secondary school | 2.41 | 5.93 | 0.02 | 0.41 | -9.23 | 14.04 | 0.685 |
| College | 9.00 | 5.23 | 0.07 | 1.72 | -1.26 | 19.26 | 0.086 |
| ‘Other’ | -1.23 | 7.20 | -0.01 | -0.17 | -15.35 | 12.89 | 0.864 |
| Ethnicity | -5.55 | 18.45 | -0.01 | -0.30 | -41.75 | 30.66 | 0.764 |
| Equivalized income | 0.00 | 0.00 | -0.01 | -0.14 | 0.00 | 0.00 | 0.889 |
| Number of additional household members with coeliac disease | 16.90 | 5.65 | 0.09 | 2.99 | 5.82 | 27.98 | 0.003 |
| Index of Multiple Deprivation Quintiles | -2.56 | 1.56 | -0.05 | -1.64 | -5.63 | 0.50 | 0.101 |
| Time since diagnosis of coeliac disease | -0.20 | 0.15 | -0.04 | -1.30 | -0.49 | 0.10 | 0.195 |
| Total number of comorbidities | 2.22 | 1.28 | 0.06 | 1.73 | -0.30 | 4.74 | 0.084 |
| (Constant) | 119.59 | 23.06 |  | 5.19 | 74.34 | 164.84 | <.001 |

**S Table 4: Regression analysis for self-reported spending on GFSF in a month (outcome variable) and self-reported prescription status (main explanatory variable) (R^2^_adj_ 0.12, p<0.001) (n=1074)**

| **Variables** | **Unstandardized coefficient** | | **Standardized Coefficients β** | **t** | **95% CI** | | **p** |
| --- | --- | --- | --- | --- | --- | --- | --- |
|  | **β** | **Standard error** |  |  | **Lower** | **Upper** |  |
| Self-report prescription status | 14.73 | 4.00 | 0.11 | 3.68 | 6.88 | 22.58 | <.001 |
| Affordability to buy GFSF |  |  |  |  |  |  |  |
| Can comfortably buy GFSF | Ref | - | - | - | - | - | - |
| Can just afford to buy GFSF | 19.20 | 1.55 | 0.13 | 3.77 | 2.79 | 8.86 | <.001 |
| Cannot always afford to buy GFSF | 2.18 | 2.23 | 0.03 | 0.98 | -2.19 | 6.54 | 0.328 |
| Sex | 9.65 | 1.50 | 0.21 | 6.42 | 6.70 | 12.59 | <.001 |
| Age | 0.07 | 0.07 | 0.05 | 1.04 | -0.06 | 0.21 | 0.298 |
| Occupation |  |  |  |  |  |  |  |
| Employed (full time, part time and self- employed)’ | Ref | - | - | - | - | - | - |
| Unemployed | 42.35 | 23.30 | 0.05 | 1.82 | -3.38 | 88.07 | 0.069 |
| Permanently sick or disabled | 7.02 | 10.72 | 0.02 | 0.66 | -14.01 | 28.05 | 0.513 |
| Fully retired from work | -0.39 | 5.54 | 0.00 | -0.07 | -11.26 | 10.49 | 0.944 |
| Looking after the home | 0.12 | 5.63 | 0.00 | 0.02 | -10.92 | 11.17 | 0.983 |
| ‘Other’ employment | -10.31 | 6.45 | -0.05 | -1.60 | -22.96 | 2.34 | 0.110 |
| Full or part-time education | -22.66 | 13.94 | -0.05 | -1.63 | -50.01 | 4.69 | 0.104 |
| Marital status |  |  |  |  |  |  |  |
| Married | Ref | - | - | - | - | - | - |
| Single | -1.83 | 5.95 | -0.01 | -0.31 | -13.51 | 9.85 | 0.759 |
| Separated, divorced or legally dissolved civil partnership | -8.40 | 6.39 | -0.04 | -1.31 | -20.93 | 4.14 | 0.189 |
| Widowed or surviving partner of civil partnership | -6.82 | 7.44 | -0.03 | -0.92 | -21.42 | 7.78 | 0.360 |
| Socioeconomic background |  |  |  |  |  |  |  |
| Higher & intermediate managerial, administrative, professional occupations | Ref | - | - | - | - | - | - |
| Supervisory or clerical, junior managerial, administrative or professional | -12.43 | 5.13 | -0.09 | -2.42 | -22.49 | -2.36 | 0.016 |
| Skilled manual | -17.25 | 8.62 | -0.07 | -2.00 | -34.16 | -0.34 | 0.046 |
| Semi or unskilled manual | -17.63 | 10.78 | -0.06 | -1.64 | -38.79 | 3.53 | 0.102 |
| State pensioners or widows (no other earner) | -1.90 | 6.82 | -0.01 | -0.28 | -15.29 | 11.49 | 0.781 |
| Casual or minimum earners | -12.32 | 11.17 | -0.04 | -1.10 | -34.23 | 9.60 | 0.270 |
| Educational level |  |  |  |  |  |  |  |
| University undergraduate or postgraduate degree) | Ref | - | - | - | - | - | - |
| Secondary school | 2.34 | 5.91 | 0.02 | 0.40 | -9.25 | 13.93 | 0.693 |
| College | 8.65 | 5.21 | 0.06 | 1.66 | -1.57 | 18.87 | 0.097 |
| ‘Other’ | -2.10 | 7.17 | -0.01 | -0.29 | -16.18 | 11.97 | 0.769 |
| Ethnicity | -7.04 | 18.38 | -0.01 | -0.38 | -43.12 | 29.03 | 0.702 |
| Equivalized income | 0.00 | 0.00 | -0.01 | -0.30 | 0.00 | 0.00 | 0.762 |
| Number of additional household members with coeliac disease | 17.15 | 5.62 | 0.09 | 3.05 | 6.11 | 28.18 | 0.002 |
| Index of Multiple Deprivation Quintiles | -2.49 | 1.55 | -0.05 | -1.60 | -5.54 | 0.56 | 0.109 |
| Time since diagnosis of coeliac disease | -0.17 | 0.15 | -0.04 | -1.15 | -0.47 | 0.12 | 0.250 |
| Total number of comorbidities | 2.35 | 1.28 | 0.06 | 1.84 | -0.16 | 4.86 | 0.066 |
| (Constant) | 112.57 | 23.08 |  | 4.88 | 67.28 | 157.87 | <.001 |

**S Table 5: Regression analysis for estimated amount spent on GFSF in a month (outcome variable) and local area prescription policy (main explanatory variable). The sample was restricted to only participants whose self-reported prescription status matched the local area prescription policy (R^2^_adj_ 0.16, p<0.001) (n=784).**

| **Variables** | **Unstandardized coefficient** | | **Standardized Coefficients β** | **t** | **95% CI** | | **p** |
| --- | --- | --- | --- | --- | --- | --- | --- |
|  | **β** | **Standard error** |  |  | **Lower** | **Upper** |  |
| Local area prescription policy | 15.20 | 1.67 | 0.31 | 9.12 | 11.93 | 18.47 | <.001 |
| Affordability to buy GFSF |  |  |  |  |  |  |  |
| Can comfortably buy GFSF | Ref | - | - | - | - | - | - |
| Can just afford to buy GFSF | 6.39 | 1.81 | 0.14 | 3.53 | 2.84 | 9.94 | <.001 |
| Cannot always afford to buy GFSF | 0.07 | 2.71 | 0.00 | 0.03 | -5.24 | 5.38 | 0.978 |
| Sex | 8.50 | 1.77 | 0.18 | 4.79 | 5.02 | 11.98 | <.001 |
| Age | 0.06 | 0.08 | 0.04 | 0.74 | -0.10 | 0.22 | 0.462 |
| Occupation |  |  |  |  |  |  |  |
| Employed (full time, part time and self- employed)’ | Ref | - | - | - | - | - | - |
| Unemployed | 7.82 | 11.80 | 0.02 | 0.66 | -15.33 | 30.98 | 0.507 |
| Permanently sick or disabled | 4.25 | 4.07 | 0.04 | 1.05 | -3.73 | 12.23 | 0.296 |
| Fully retired from work | -2.60 | 2.08 | -0.06 | -1.25 | -6.69 | 1.48 | 0.211 |
| Looking after the home | 1.03 | 2.14 | 0.02 | 0.48 | -3.17 | 5.24 | 0.629 |
| ‘Other’ employment | 2.16 | 2.56 | 0.03 | 0.85 | -2.86 | 7.18 | 0.398 |
| Full or part-time education | -2.01 | 6.73 | -0.01 | -0.30 | -15.22 | 11.20 | 0.765 |
| Marital status |  |  |  |  |  |  |  |
| Married | Ref | - | - | - | - | - | - |
| Single | -0.84 | 2.38 | -0.01 | -0.35 | -5.51 | 3.83 | 0.725 |
| Separated, divorced or legally dissolved civil partnership | -4.50 | 2.40 | -0.07 | -1.87 | -9.22 | 0.22 | 0.062 |
| Widowed or surviving partner of civil partnership | -3.41 | 2.77 | -0.04 | -1.23 | -8.84 | 2.03 | 0.219 |
| Socioeconomic background |  |  |  |  |  |  |  |
| Higher & intermediate managerial, administrative, professional occupations | Ref | - | - | - | - | - | - |
| Supervisory or clerical, junior managerial, administrative or professional | -3.64 | 2.08 | -0.08 | -1.75 | -7.71 | 0.44 | 0.080 |
| Skilled manual | -2.51 | 3.24 | -0.03 | -0.78 | -8.88 | 3.85 | 0.438 |
| Semi or unskilled manual | 0.60 | 4.09 | 0.01 | 0.15 | -7.43 | 8.63 | 0.884 |
| State pensioners or widows (no other earner) | -1.31 | 2.62 | -0.03 | -0.50 | -6.46 | 3.84 | 0.618 |
| Casual or minimum earners | -3.72 | 4.50 | -0.03 | -0.83 | -12.55 | 5.11 | 0.409 |
| Educational level |  |  |  |  |  |  |  |
| University undergraduate or postgraduate degree) | Ref | - | - | - | - | - | - |
| Secondary school |  | 2.35 | 0.03 | 0.66 | -3.05 | 6.17 | 0.507 |
| College | 0.52 | 2.07 | 0.01 | 0.25 | -3.55 | 4.59 | 0.803 |
| ‘Other’ | 1.73 | 2.87 | 0.02 | 0.60 | -3.90 | 7.35 | 0.548 |
| Ethnicity | 0.55 | 7.72 | 0.00 | 0.07 | -14.61 | 15.71 | 0.943 |
| Equivalized income | 0.00 | 0.00 | 0.00 | -0.06 | 0.00 | 0.00 | 0.954 |
| Number of additional household members with coeliac disease | 11.35 | 2.49 | 0.15 | 4.55 | 6.45 | 16.24 | <.001 |
| Index of Multiple Deprivation Quintiles | 0.37 | 0.62 | 0.02 | 0.60 | -0.84 | 1.59 | 0.549 |
| Time since diagnosis of coeliac disease | 0.00 | 0.06 | 0.00 | -0.01 | -0.12 | 0.12 | 0.991 |
| Total number of comorbidities | -0.21 | 0.49 | -0.02 | -0.43 | -1.18 | 0.76 | 0.670 |
| (Constant) | 19.11 | 9.63 |  | 1.99 | 0.21 | 38.01 | 0.047 |
